# Supplementary material for: A lung-specific mutational signature enables inference of viral and bacterial respiratory niche
Source: Microb Genom. 2023 May 15;9(5):mgen001018. doi: 10.1099/mgen.0.001018 (PMC10272861; doi:10.1099/mgen.0.001018)
Supplement: Supplementary material 4 [file mgen-9-1018-s001.pdf]

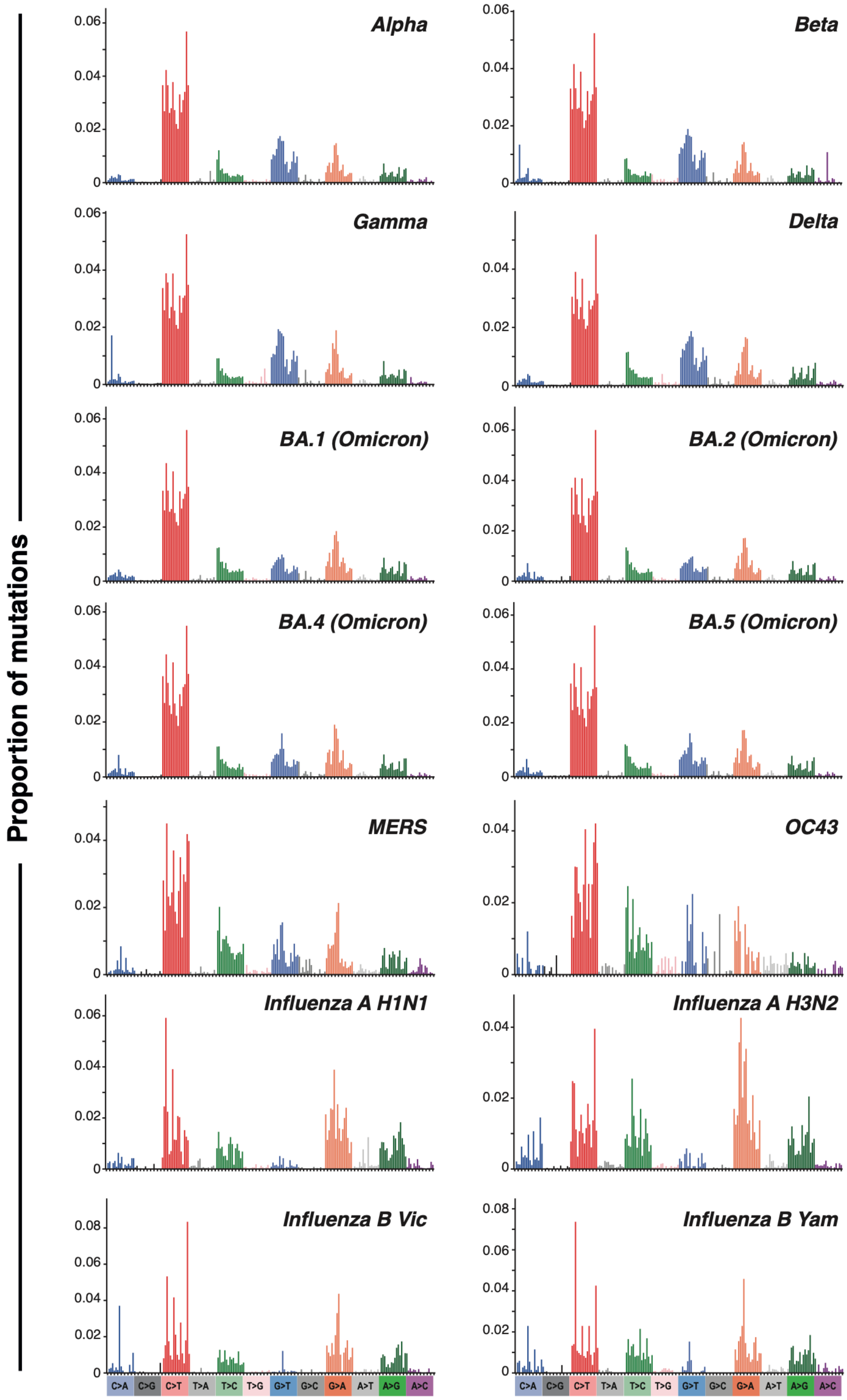

Figure S1. SBS spectra of respiratory viruses. Spectra are rescaled by genomic context availability.

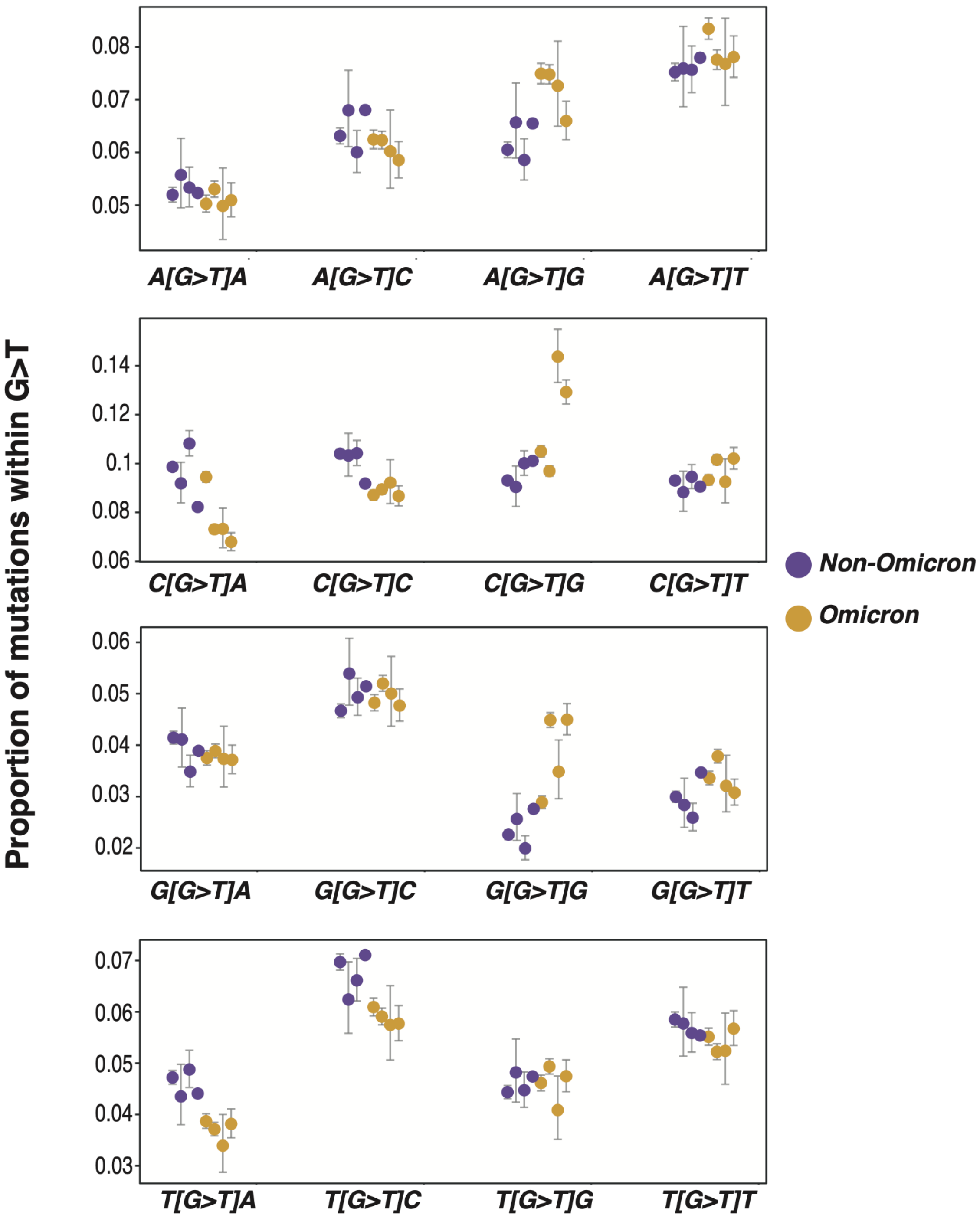

Figure S2. Contextual mutational patterns within G>T are similar between Omicron and non-Omicron lineages. The proportion of G>T mutations within each of the 16 surrounding contexts is plotted for the non-Omicron lineages (Alpha, Beta, Gamma and Delta) and the Omicron lineages (BA.1, BA.2, BA.4, BA.5). Error bars show confidence intervals, calculated as the Wilson score interval using the number of G>T mutations as the number of trials and proportion of G>T mutations within the context as the success proportion. We do not observe a conserved significant difference between non-Omicron and Omicron lineages within any context.

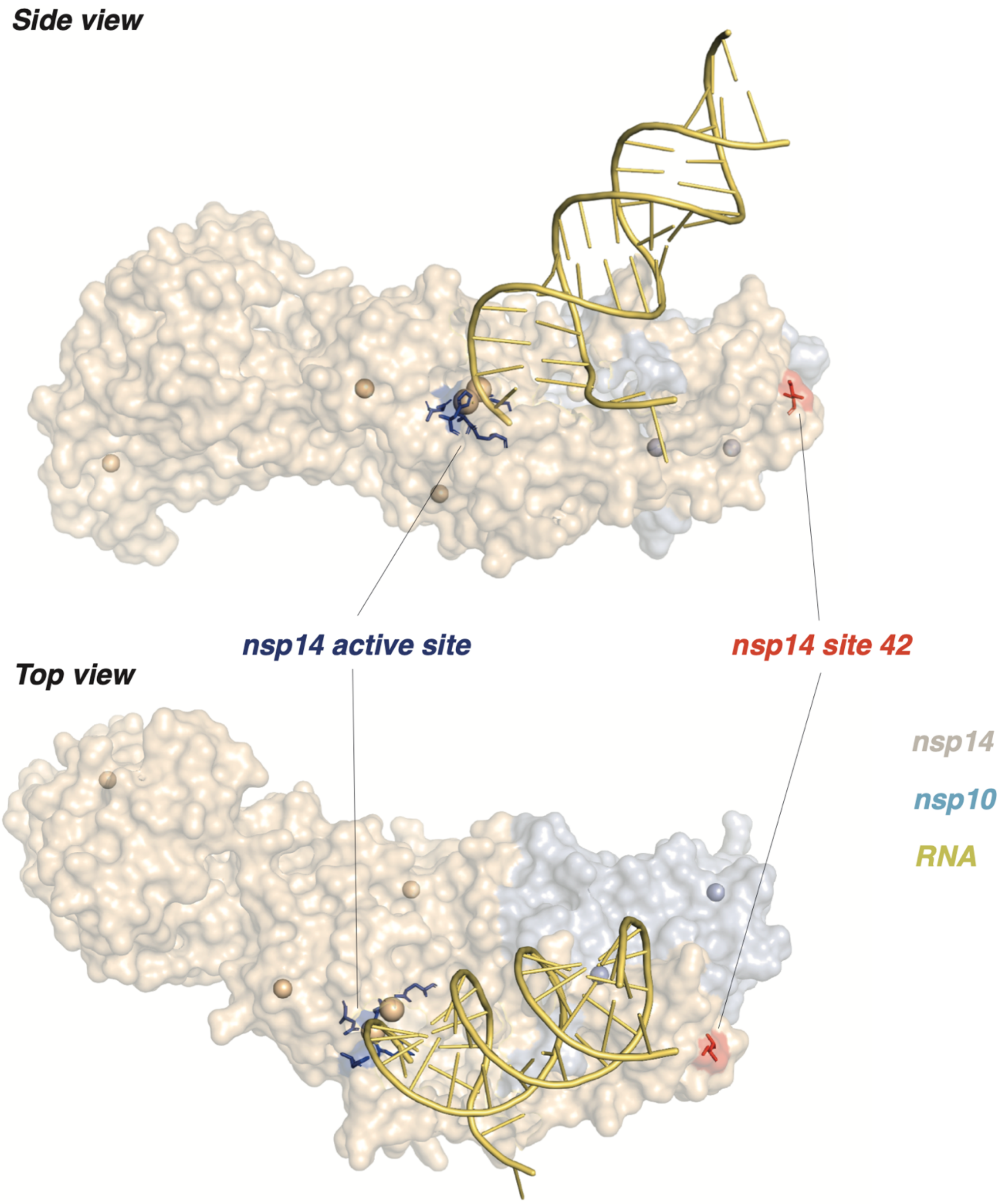

Figure S3. nsp14 mutation I42V is distal to the active site. The structure of the SARS-CoV-2 nsp14 (coloured light brown) and nsp10 (coloured light blue) complex bound to RNA (coloured yellow) is shown (PDB accession 7N0B). The SARS-CoV-2 proteins are shown as surfaces. The nsp14 active site (residues D90, E92, E191, H268 and D273) responsible for cleavage of misincorporated nucleotides is shown as sticks in dark blue. Site 42 in nsp14 which is mutated from isoleucine to valine in Omicron is shown as sticks in red.
